# Quantifying Stock Return Distributions in Financial Markets

**DOI:** 10.1371/journal.pone.0135600

**Published:** 2015-09-01

**Authors:** Federico Botta, Helen Susannah Moat, H. Eugene Stanley, Tobias Preis

**Affiliations:** 1 Centre for Complexity Science, University of Warwick, Coventry, CV4 7AL, United Kingdom; 2 Data Science Lab, Behavioural Science, Warwick Business School, University of Warwick, Coventry, CV4 7AL, United Kingdom; 3 Center for Polymer Studies and Department of Physics, Boston University, Boston, MA 02215, United States of America; Peking University, CHINA

## Abstract

Being able to quantify the probability of large price changes in stock markets is of crucial importance in understanding financial crises that affect the lives of people worldwide. Large changes in stock market prices can arise abruptly, within a matter of minutes, or develop across much longer time scales. Here, we analyze a dataset comprising the stocks forming the Dow Jones Industrial Average at a second by second resolution in the period from January 2008 to July 2010 in order to quantify the distribution of changes in market prices at a range of time scales. We find that the tails of the distributions of logarithmic price changes, or returns, exhibit power law decays for time scales ranging from 300 seconds to 3600 seconds. For larger time scales, we find that the distributions tails exhibit exponential decay. Our findings may inform the development of models of market behavior across varying time scales.

## Introduction

Complex movements in stock market prices affect the personal fortunes of people around the globe [1–5]. An ability to more accurately quantify and predict such changes would allow us to gain more insights into how financial crises arise [6] and provide greater empirical basis for the development of theories of financial market behavior [7–13].

The financial markets were however one of the earliest sources of large scale datesets on human behaviour, where such data have recently become the focus of the new field of computational social science [14–24]. A vast amount of data on financial decisions made in stock markets is therefore available [25–29]. Previous studies have shown that distributions of returns observed in empirical data are consistent with power law decay [30–42], in contrast with widely used models that assume Gaussian behavior of these returns. Power law behavior has also been observed in other economical and financial sectors of society [43, 44].

Changes in stock market prices can occur at a range of different time scales. Here, we analyze a large dataset of stocks forming the Dow Jones Industrial Average (DJIA) at a second-by-second resolution for a range of different time scales in order to quantify the distribution of returns. We provide evidence that while the distribution of returns exhibits power law behavior at small time scale, exponential behavior is observed at larger time scales. We find analogous results when restricting our analysis to volatile trading periods. Our findings could help to gain insight into changes in stock market prices in shorter periods and longer periods and provide further empirical basis for the development of new models of market behavior.

## Results

**Fig 1:**
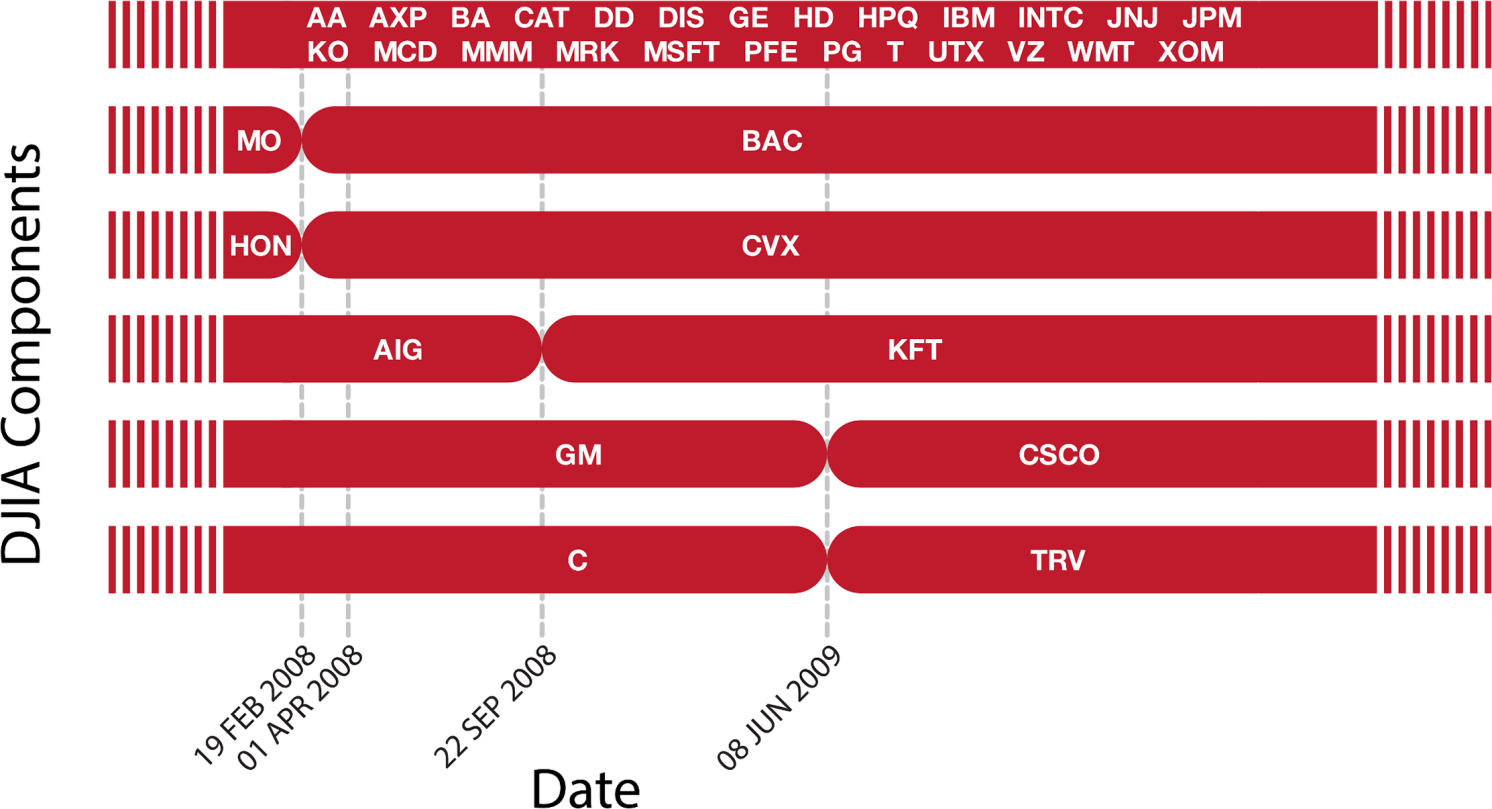
Components of the DJIA. Here we depict the components of the DJIA in the time period between 02 January 2008 to 30 July 2010. Dashed vertical lines correspond to changes in the stocks forming the DJIA. In our analysis, we focus on the 25 stocks that were part of the DJIA during the period of analysis. Stocks are labelled using ticker symbols that uniquely identify the company name, as used by the stock exchange.

The DJIA is a U.S. benchmark index that consists of 30 different stocks. For all 30 stocks, we retrieve price time series with a second by second resolution from the Trade and Quote (TAQ) database provided by Wharton Research Data Services (WRDS). Our dataset covers the period from 2 January 2008 to 30 July 2010 comprising a total of 647 trading days. Fig 1 shows the various components of the DJIA. As five stocks were replaced during this period, we focus on the 25 components that were consistently part of the DJIA between 02 January 2008 and 30 July 2010.

We define returns as the relative logarithmic change in price of a given stock *i* at a given time *t*:

$$r_{i}\left(t\right)=\log\left(p_{i}\left(t+\Delta t\right)\right)-\log\left(p_{i}\left(t\right)\right)\;i=1,\ldots ,25$$

where Δ*t* is the time lag between price observations. As a trading day starts at 9:30 and ends at 16:00 local time, Δ*t* is constrained to be at most 6 hours and 30 minutes.

**Fig 2:**
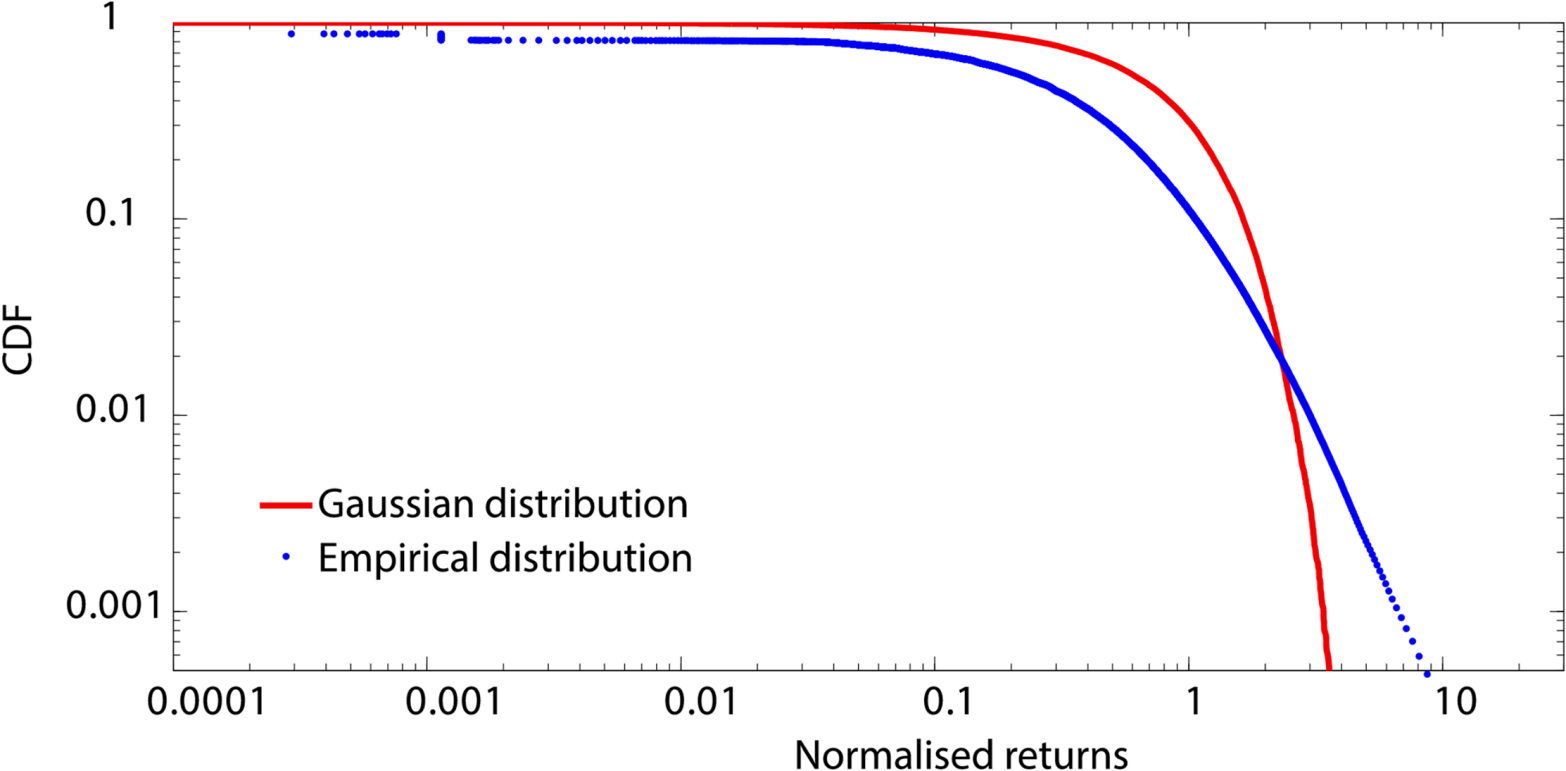
Empirical distribution of normalised returns for *American Express*. We build returns distributions for the 25 stocks of the DJIA for different time lags across the full period of analysis. We standardize each distribution by subtracting the mean return from each observation and dividing by the standard deviation. We depict in blue the cumulative distribution function of the positive component of the return distributions for *American Express* for a time lag of 300 seconds. We depict in red the positive tail of a Gaussian distribution with mean zero and standard deviation one. We observe a strong deviation of the empirical distribution from the Gaussian distribution. Instead, visual inspection of the distribution tail reveals consistency with a linear relationship on a log-log scale. This provides initial evidence for possible power law behavior at this time scale.

We compute the standardized distribution of the returns for the 25 components of the DJIA that we consider. We conduct separate analyses of the cumulative distribution function (CDF) of the positive and negative component of the distribution of returns. Fig 2 depicts the positive CDF for *American Express* for Δ*t* = 300 seconds and compares this to a Gaussian distribution. Note that the empirical distribution strongly deviates from the Gaussian distribution and provides initial evidence for power law behavior. We perform a statistical analysis to check the consistency of the tails of the empirical distributions with power law behavior across different time scales, as proposed by Clauset, Shalizi and Newman [45] and detailed in the Methods section.

### Changes in power law behavior as Δ*t* increases

**Fig 3:**
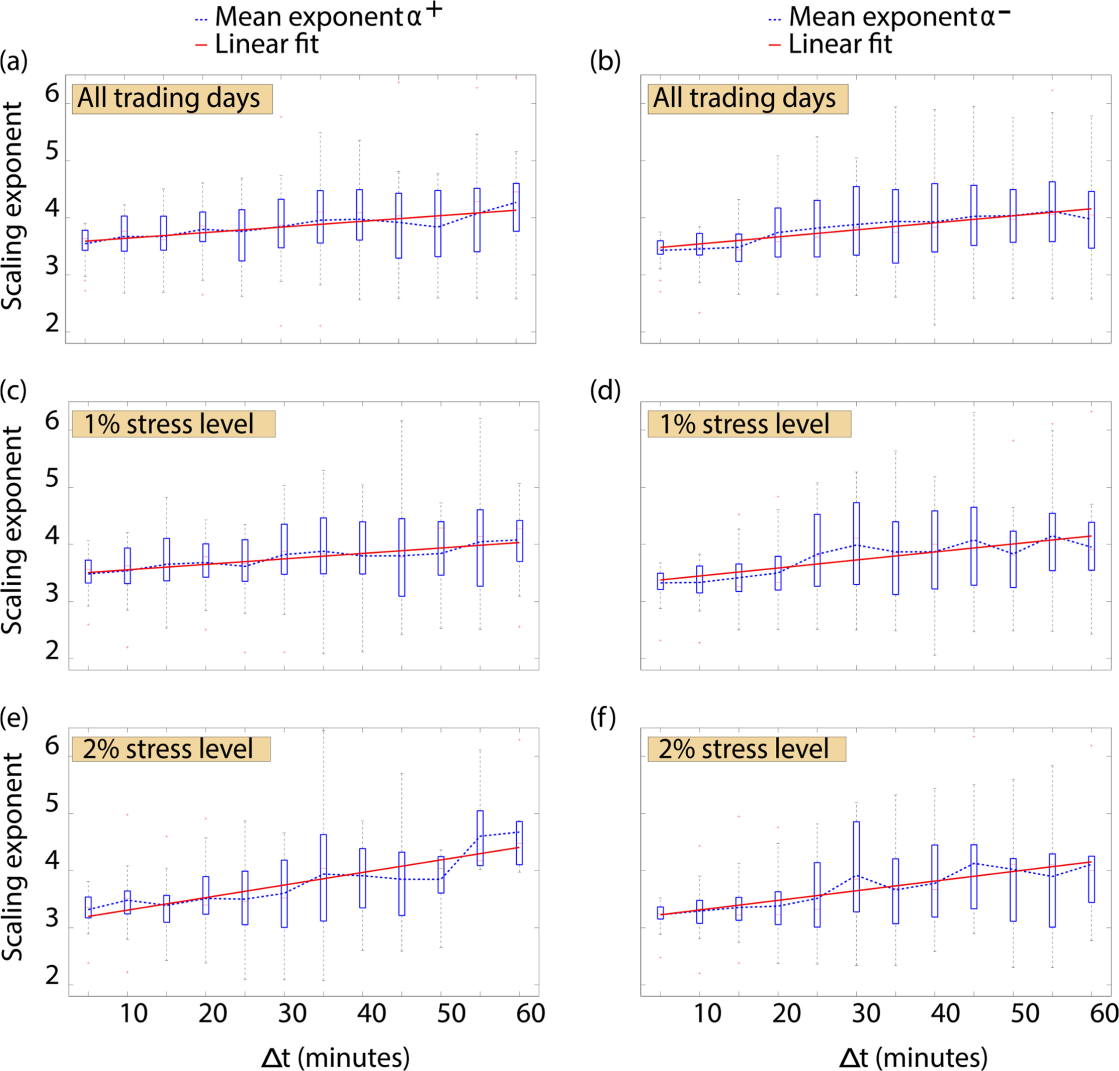
Relationship between Δ*t* and the scaling exponent for the empirical tails of return distributions. (a) We investigate the relationship between the time lag between price observations used to build the returns distribution and the scaling exponents of the tails of distributions. We consider here the tails of the positive component of the distributions obtained when analyzing all trading days present in our dataset. We find that the mean scaling exponent increases as Δ*t* increases (Adjusted *R* ^2^ = 0.802, *N* = 12, *p* < 0.001, ordinary least squares regression) (b) In a similar fashion, we observe that when analyzing all trading days the mean scaling exponent for the tail of the negative component of the distributions increases with the time lag (Adjusted *R* ^2^ = 0.839, *N* = 12, *p* < 0.001, ordinary least squares regression) (c) We now restrict our analysis to trading days on which the prices of stocks have changed by more than 1%. We find that the mean scaling exponent of positive tails consistent with power law behavior increases with Δ*t* (Adjusted *R* ^2^ = 0.856, *N* = 12, *p* < 0.001, ordinary least squares regression) (d) Under 1% stress, an increase in the time lag Δ*t* results again in an increase of the mean scaling exponent for the tails of the negative returns distributions (Adjusted *R* ^2^ = 0.729, *N* = 12, *p* < 0.001, ordinary least squares regression) (e) We now perform the same analysis for days on which the prices of stocks have changed by more than 2%. The mean scaling exponent for the tails of the positive component of the distributions again shows an increase with increasing Δ*t* (Adjusted *R* ^2^ = 0.782, *N* = 12, *p* < 0.001, ordinary least squares regression) (f) Similarly, the mean scaling exponent for the tails of negative returns distributions at the 2% stress level increases as the time lag Δ*t* between price observations increases (Adjusted *R* ^2^ = 0.836, *N* = 12, *p* < 0.001, ordinary least squares regression).

A power law probability distribution is a probability distribution in which the probability of an event decays as a negative power of the event. The distribution function is characterized by a scaling exponent. Distributions of returns typically exhibit power law decay in the tail of the distribution. Here, we want to understand how the exact nature of power law behavior depends on the time lag between price observations. We analyze all 25 stock price time series and use a time lag Δ*t* ranging from 300 to 3,600 seconds. We investigate how the scaling exponent changes as a function of the time lag between price observations. We depict the exponent for the tails of the positive (denoted as *α* ^+^; Fig 3a) and negative (denoted as *α* ^−^; Fig 3b) returns distributions obtained when analyzing data from all trading days. For both positive and negative tails, we find that the mean scaling exponent increases with the time lag Δ*t* (*α* ^+^: Adjusted *R* ^2^ = 0.802, *N* = 12, *p* < 0.001, ordinary least squares regression; *α* ^−^: Adjusted *R* ^2^ = 0.839, *N* = 12, *p* < 0.001, ordinary least squares regression):

$$\alpha^{+}=0.010\left(\pm 0.001\right)\Delta t+3.54\left(\pm 0.05\right)$$

$$\alpha^{-}=0.012\left(\pm 0.001\right)\Delta t+3.42\left(\pm 0.06\right)$$

We find similar slopes for the positive and negative tails, which suggests that both exponents *α* ^+^ and *α* ^−^ vary in a similar fashion as a function of the time lag Δ*t*. Our results suggest that the probability of finding large price changes is underestimated by a Gaussian distribution and better quantified by a power law distribution, in line with a range of findings reported in the field of econophysics [30–42]. Previous findings for US markets have highlighted that stock returns may follow an inverse cubic law [31]. The analysis of different stock markets, such as the Warsaw Stock Exchange in Poland or the Australian Stock Exchange, has uncovered different power law regimes deviating from the inverse cubic law [38, 39]. By selecting appropriate cutoff values in the distributions under analysis, stocks from the Mexican Stock Market index exhibit a power law decay close to an inverse cubic law [40]. Analogous results have also been observed when analysing daily returns in Chinese stock markets [46, 47].

It remains unclear, however, whether these findings hold for subsets of the price time series in which extreme price movements occur. We therefore restrict our analysis to price observations recorded on trading days on which the corresponding stock gained or lost more than 1% on a daily basis. We refer to this as a stress level of 1%. Fig 3c and 3d depict the relationship between the power law exponents and the time lag Δ*t* between price observations on trading days on which the market experienced a stress level of at least 1%.

We again find that an increase in Δ*t* results in an increase of the mean scaling exponent (*α* ^+^: Adjusted *R* ^2^ = 0.856, *N* = 12, *p* < 0.001, ordinary least squares regression; *α* ^−^: Adjusted *R* ^2^ = 0.729, *N* = 12, *p* < 0.001, ordinary least squares regression):

$$\alpha^{+}=0.010\left(\pm 0.001\right)\Delta t+3.46\left(\pm 0.04\right)$$

$$\alpha^{-}=0.014\left(\pm 0.003\right)\Delta t+3.31\left(\pm 0.09\right)$$

We notice a strong similarity between the relationship between the scaling exponent and Δ*t* in this scenario and in the scenario where we consider all trading days. In a parallel analysis, we consider a 2% stress level (Fig 3e and 3f). We find that the mean scaling exponent increases with the time lag Δ*t* between price observations (*α* ^+^: Adjusted *R* ^2^ = 0.782, *N* = 12, *p* < 0.001, ordinary least squares regression; *α* ^−^: Adjusted *R* ^2^ = 0.836, *N* = 12, *p* < 0.001, ordinary least squares regression):

$$\alpha^{+}=0.022\left(\pm 0.003\right)\Delta t+3.09\left(\pm 0.13\right)$$

$$\alpha^{-}=0.017\left(\pm 0.002\right)\Delta t+3.14\left(\pm 0.08\right)$$

At a stress level of 3%, we again observe that the scaling exponent increases as we increase the time lag Δ*t* (*α* ^+^: Adjusted *R* ^2^ = 0.573, *N* = 12, *p* < 0.05, ordinary least squares regression; *α* ^−^: Adjusted *R* ^2^ = 0.458, *N* = 12, *p* < 0.05, ordinary least squares regression):

$$\alpha^{+}=0.066\left(\pm 0.017\right)\Delta t+2.04\left(\pm 0.61\right)$$

$$\alpha^{-}=0.016\left(\pm 0.005\right)\Delta t+2.91\left(\pm 0.19\right)$$

### Evidence of exponential decay at larger values of Δ*t*

**Fig 4:**
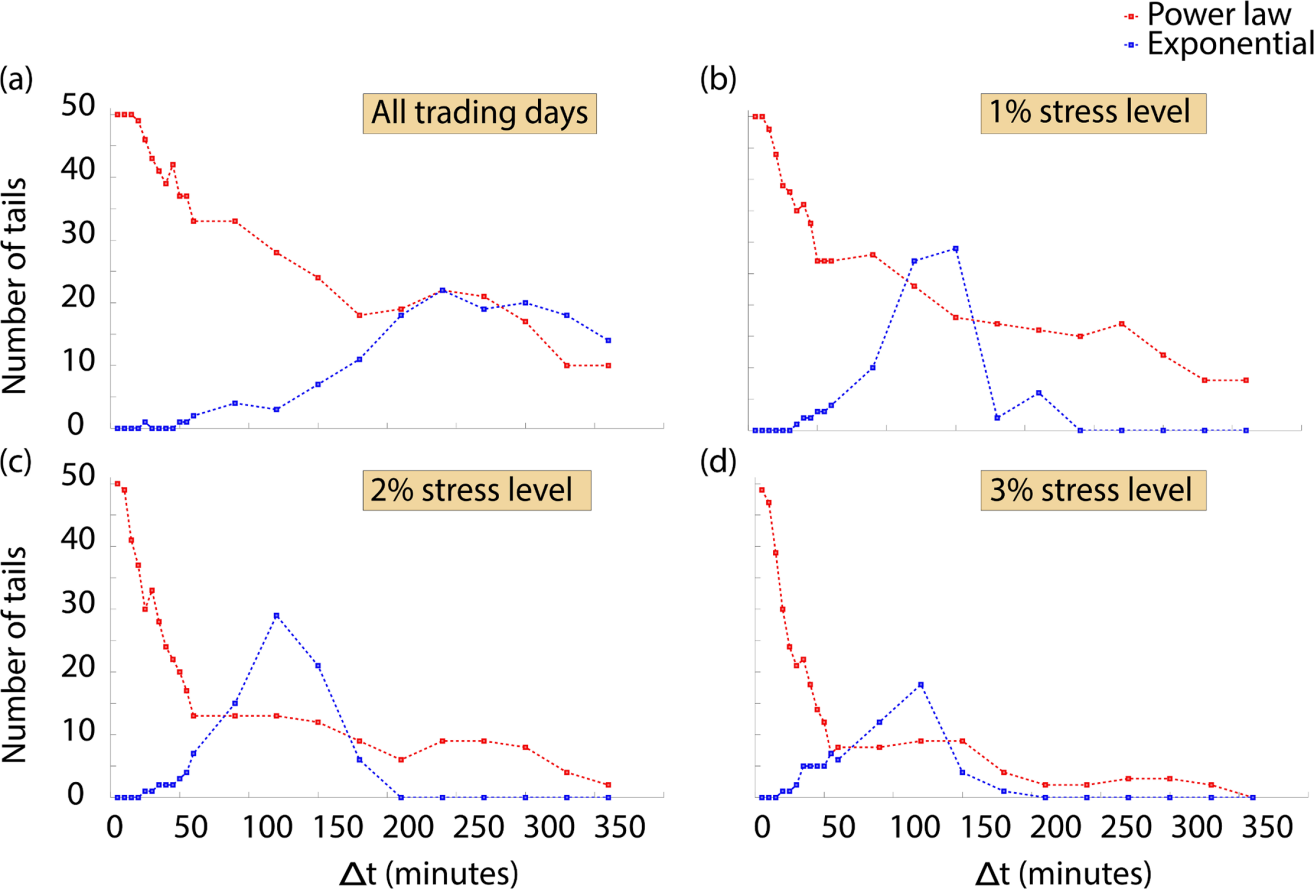
Consistency of empirical returns distributions with power law and exponential decay. (a) For Δ*t* > 60 minutes, we note a decrease in the number of tails consistent with power law decay. We investigate whether the tails of the returns distributions are consistent with power law behavior or exponential decay using the Kolmogorov-Smirnov statistic, as described in the methods section. We first consider all trading days present in our dataset. At short time scales, we observe that the tails of most empirical distributions are consistent with power law behavior. As we increase the time lag, the number of tails consistent with power law behavior decreases and we see an increase in the number of tails of returns distributions that are consistent with exponential decay. We depict here the overall number of tails, both for the positive and negative returns distributions, for the 25 components of the DJIA. (b) We consider transaction days on which the prices of stocks have changed by more than 1%. We refer to this as a stress level of 1%. In this scenario, the number of tails consistent with power law decreases more sharply. Consistency with exponential decay appears when Δ*t* is roughly 2 hours. (c) In a similar fashion, when we consider a stress level of 2%, we again observe a sharp decrease in the number of distributions consistent with power law behavior. We also find an increase in the number of tails consistent with exponential decay again when Δ*t* is roughly 2 hours. (d) Under a stress level of 3%, the number of empirical distributions consistent with power law behavior decreases more quickly than in the other scenarios. The number of tails consistent with exponential decay peaks at a lower number than in other scenarios, but is again highest when Δ*t* is roughly two hours, similar to other scenarios.

We observe that for Δ*t* > 60 minutes the number of tails consistent with power law behavior decreases (Fig 4a). We investigate this change in behavior at a range of time scales and analyze whether we start to observe consistency with exponential decay. Exponential decay has already been observed in daily returns of stocks from the National Stock Exchange in the Indian stock market [48].

Fig 4a depicts how the number of distributions consistent with either power law behavior or exponential decay varies with Δ*t*. At small time scales, the tail of most distributions is consistent with power law behavior. As we increase the time lag between price observations, we observe an increase in the number of tails consistent with exponential decay. At the 1% stress level, the decrease in the number of tails consistent with power law is sharper and we find a peak in the number of tails consistent with exponential decay when Δ*t* is roughly 2 hours (Fig 4b). As we increase the stress level, we find that the number of tails consistent with power law behavior decreases even more sharply. The number of tails consistent with exponential decay exhibits a peak at similar time scales, but peaks at a lower number than observed at the 1% stress level (Fig 4c and 4d).

## Conclusions

Large changes in stock market prices can occur at a range of time scales, arising within minutes or developing across longer time scales. Our findings provide evidence that in different scenarios the scaling exponent of those distributions consistent with power law behavior increases with the time lag between price observations. As this time lag increases, we observe that the number of return distributions consistent with power law behavior decreases sharply. At a time lag of roughly two hours, we also find an increase in the number of distributions which are consistent with exponential decay. Our results are consistent with the hypothesis that changes in stock market prices have different behaviors at different time scales. We observe that these results hold in different scenarios of the market, both when we consider all trading days, but also when restricting our analysis to scenarios with different stress levels. We suggest that our analysis may provide further empirical insights for the development of models of market behavior.

## Methods

To check the consistency of the tails of observed empirical distributions with power law behavior, we follow the procedure proposed in Ref. [45], which we summarise again here.

A power law is a distribution of the form:

$$p\left(x\right)=\frac{\alpha -1}{x_{\text{min}}}{\left(\frac{x}{x_{\text{min}}}\right)}^{-\alpha }$$

where *α* is the scaling exponent. We require *α* > 1 for this to be a Probability Distribution Function (PDF). *x*_*min*_ is the lower bound of the power law behavior. We estimate the scaling exponent *α* using the maximum likelihood estimator (MLE). Assuming we have *n* observations of *x*_*i*_(*i* = 1, …, *n*) which are independent and identically distributed random variables, the likelihood function, which represents the probability of observing the data given the parameter, is given by:

$$p\left(x|\alpha \right)=\prod_{i=1}^{n}\frac{\alpha -1}{x_{\text{min}}}{\left(\frac{x_{i}}{x_{\text{min}}}\right)}^{-\alpha }$$

We then maximise this probability to find the MLE estimator for the scaling exponent:

$$\hat{\alpha }=1+n\;[\sum_{i=1}^{n}\ln\frac{x_{i}}{x_{\text{min}}}]$$

We measure distances between distribution using the Kolmogorov-Smirnov statistic (KS statistic):

$$D=max_{x\geq x_{min}}\left|E\left(x\right)-F\left(x\right)\right|$$

where *E*(*x*) is the empirical CDF and *F*(*x*) is the best fit of the data. We determine the lower bound *x*_min_ by choosing the value that minimizes the distance between the empirical distribution and the fitted distribution as measured by the KS statistic.

Once we have determined the lower bound *x*_min_ and the scaling exponent *α*, we then check the consistency of the hypothesis of power law behavior in the observed empirical distributions. We construct the empirical tails choosing a bin size such that we have 1,000 data points in each tail. We then compare the KS statistic observed for the empirical data when compared to a fitted power law distribution with the KS statistic obtained for the synthetic data when compared to a fitted power law distribution. We obtain a *p*-value by counting the number of times that the synthetic KS statistic is larger than the empirical KS statistic. We generate 1,000 synthetic data sets and make the conservative choice of accepting our hypothesis of consistency with power law behavior if the *p*-value is larger than 0.1.

To determine whether the distribution is consistent with exponential decay, we perform a parallel analysis fitting the data to an exponential distribution instead of a power law probability distribution. We then generate synthetic data from the fitted distribution in the same manner as previously described. We evaluate whether our data are consistent with exponential decay by comparing the empirical data to the synthetic data using KS statistics as described above.
